# Diagnostic Performance of [^18^F]Fluorocholine and [^68^Ga]Ga-PSMA PET/CT in Prostate Cancer: A Comparative Study

**DOI:** 10.3390/jcm9072308

**Published:** 2020-07-21

**Authors:** Zeinab Paymani, Taryn Rohringer, Reza Vali, Wolfgang Loidl, Nafiseh Alemohammad, Hans Geinitz, Werner Langsteger, Mohsen Beheshti

**Affiliations:** 1 Research Center for Nuclear Medicine, Shariati Hospital, Tehran University of Medical Sciences, Tehran 1411713135, Iran; paymaniz@yahoo.com; 2 Department of Nuclear Medicine & Endocrinology, PET-CT Center LINZ, St. Vincent’s Hospital, Ordensklinikum, 4020 Linz, Austria; werner.langsteger@meduniwien.ac.at; 3 Department of Diagnostic Imaging, the Hospital for Sick Children, University of Toronto, Toronto, ON M5G 1X8, Canada; taryn.rohringer@mail.utoronto.ca (T.R.); reza.vali@sickkids.ca (R.V.); 4 Department of Urology, St. Vincent’s Hospital, Ordensklinikum, 4020 Linz, Austria; wolfgang.loidl@ordensklinikum.at; 5 Department of Mathematics and Computer Science, Shahed University, Tehran, 3319118651, Iran; n.alemohammad@shahed.ac.ir; 6 Department of Radiation-Oncology, Krankenhaus Barmherzige Schwestern, Ordensklinikum, 4020 Linz, Austria; hans.geinitz@ordensklinikum.at; 7 Department of Nuclear Medicine, Medical University of Vienna, 1090 Vienna, Austria; 8 Department of Nuclear Medicine, University Hospital, RWTH University, 52074 Aachen, Germany; 9 Department of Nuclear Medicine, Paracelsus Medical University, 5020 Salzburg, Austria

**Keywords:** Prostate cancer, Positron emission tomography/computed tomography (PET/CT), [^68^Ga]Ga-PSMA, [^18^F]Fluorocholine

## Abstract

The current study endeavored to closely compare the detection rate of 68-Gallium labelled prostate-specific membrane antigen ([^68^Ga]Ga-PSMA) versus [^18^F]Fluorocholine in men with prostate cancer (PC), to investigate the benefits and pitfalls of each modality in the setting of various patient characteristics. We retrospectively analyzed 29 biopsy-proven PC patients in two categories, staging and restaging, who underwent both scans within a maximum of 30 days of each other. Variables including patient demographics, prostate specific antigen (PSA) level, Gleason score, clinical course, and following treatments were recorded. The number and location of suspicious lesions as well as uptake values were noted. A total of 148 suspicious lesions were detected, of which 70.9% (105/148) were concordantly visualized in both imaging modalities. [^68^Ga]Ga-PSMA positron emission tomography/computed tomography (PET/CT) revealed a higher number of metastatic lesions per patients (91% vs 78%). The mean of maximum standardized uptake value (SUV max) in concordant lesions was significantly higher in [^68^Ga]Ga-PSMA compared to [^18^F]Fluorocholine PET/CT (14.6 ± 8.44 vs. 6.9 ± 3.4, *p* = 0.001). Discordant lesions were detected by both modalities, but more frequently by [^68^Ga]Ga-PSMA PET/CT (20.3% in [^68^Ga]Ga-PSMA versus 8.8% by [^18^F]Fluorocholine PET/CT). In patients with PSA levels below 1.0 ng/mL and <2.0 ng/mL, [^18^F]Fluorocholine PET/CT detection rate was half (57% and 55%, respectively) that of [^68^Ga]Ga-PSMA PET/CT. Tumor, nodes and metastases (TNM) staging, and subsequently patient management, was only influenced in 4/29 patients (14%), particularly by [^68^Ga]Ga-PSMA PET/CT with PSA values under 0.5 ng/mL. [^68^Ga]Ga-PSMA PET/CT revealed superior diagnostic performance to [^18^F]Fluorocholine PET/CT in staging and restaging of PC patients, especially in cases with low PSA levels. However, in a few hormone resistant high-risk PC patients, [^18^F]Fluorocholine PET/CT may improve overall diagnostic accuracy.

## 1. Introduction

Prostate cancer (PC) is the second most common malignancy and the fifth leading cause of cancer death in men, with more than 350,000 deaths estimated globally in 2018 [1] Regarding the heterogeneous nature of the disease, accurate staging and precise localization of the lesions by proper imaging modalities significantly affects treatment selection and improves outcomes for each patient [2,3]. Despite the effectiveness of current therapeutic options, biochemical and symptomatic recurrence is common among PC patients 5 to 10 years after curative therapy [4,5]. For staging disease in patients diagnosed with intermediate- to high-risk prostate cancer, as well as those with biochemical recurrence (BCR), current guidelines recommend computed tomography (CT) of the abdomen and pelvis, abdominopelvic magnetic resonance imaging (MRI), and/or whole-body bone scintigraphy [6,7,8]. Though detection and accurate localization of recurrence is necessary for further effective management, conventional imaging modalities lack sufficient sensitivity, especially in cases with low prostate-specific antigen (PSA) values [7,9,10,11]. They additionally have been demonstrated to frequently under-stage metastases [6]. For example, CT and MRI may misinterpret small lesions under 8–10 mm in diameter, and bone scintigraphy suffers from inadequate sensitivity for the detection of osteolytic lesions and may misdiagnose benign degeneration as osteoblastic malignant lesions [6,10,11].

Alternative molecular imaging modalities are currently utilized. Radio-labeled choline is one of the most studied positron emission tomography/computed tomography (PET/CT) radiotracers in the assessment of prostate cancer [12,13]. Choline is a precursor for the cell membrane component phosphatidylcholine, and has been used as a marker of proliferation in detection of cancer cells [6]. Both [^11^C]Choline and [^18^F]Fluorocholine have been utilized and shown to have similar efficacy [12,13]. Radiolabeled-choline (i.e., ^11^C-Choline) PET/CT is currently FDA-approved for restaging of prostate cancer in the context of biochemical relapse following radical prostatectomy and has been demonstrated in the literature to have merit in general for BCR [6]. This modality has also been shown to be useful for initial staging of high-risk patients and restaging of prostate cancer recurrence [13,14,15]. However, it suffers from unsatisfactory sensitivity, particularly at prostate-specific antigen (PSA) levels below 2 ng/mL [16,17].

Prostate-specific membrane antigen (PSMA), a surface antigen highly expressed in PC, has been more recently targeted for diagnostic and therapeutic strategies. Promising results were reported for PET/CT using PSMA-ligands, especially in early biochemical recurrence and low PSA level [18,19,20]. Furthermore, 68-Gallium labelled prostate-specific membrane antigen ([^68^Ga]Ga-PSMA) PET/CT successfully changed clinical management of the disease in over 50% of patients with BCR [21]. There is evidence in the literature that [^68^Ga]Ga-PSMA PET/CT was able identify recurrent lesions in 43.8% of patients with negative scans on [^18^F]Fluorocholine PET/CT imaging [22].

Therefore, investigating the use of each of these modalities in identical patients provides a unique opportunity to directly compare efficacy and determine the most appropriate modality in patients with various characteristics. In this study, we compared diagnostic outcomes of [^68^Ga]Ga-PSMA and [^18^F]Fluorocholine PET/CT in the same group of PC patients referred for staging or restaging.

## 2. Materials and Methods

### 2.1. Patients

This study was performed according to the 1964 Declaration of Helsinki and its later amendments or comparable standards, and was approved by our institutional review board. Informed consent for using the imaging data for scientific purposes was obtained from all patients in the context of performing the PET/CT examinations.

Twenty-nine biopsy proven PC patients were enrolled in this retrospective study. The purpose of scanning was staging in 11 (38%) patients and restaging in the remaining 18 (62%). All patients underwent both scans consecutively within an interval of less than four weeks between each other. Patients who received PET/CT scanning at our institution between April 2015 and December 2017 were retrospectively studied. In order to be included, patients had to be diagnosed with prostate cancer and had to have underwent both [^18^F]Fluorocholine and [^68^Ga]Ga-PSMA PET/CT scans for either staging or restaging within 30 days of each other, without any specific changes in concomitant prostate cancer treatment between the PET/CT examinations. Furthermore, clinical data including PSA level, Gleason score, and clinical course of disease and treatment following scanning had to be available. Patients with a history of a second malignancy were excluded from the study.

### 2.2. Study Agents

The [^68^Ga]Ga-PSMA was prepared using a lyophilized PSMA-11 Sterile Cold Kit (ANMI SA, Liege, Belgium), a commercial Good Manufacturing Practice (GMP)manufactured ^68^Ge/^68^Ga generator. The feasibility of this preparation method in clinical practice is reported in our previous study [23]. Radioactivity of 1.8–2.2 MBq per kilogram of bodyweight of [^68^Ga]Ga-PSMA was administered to study participants by intravenous bolus injection.

Fluorine-18 Fluoromethyldimethyl-2-hydroxyethyl-ammonium ([^18^F]Fluorocholine; IASOCHOLINE^®^; IASON GmbH, Graz, Austria) was used for the second PET/CT imaging. Radioactivity of 4 MBq per kilogram of body weight of [^18^F]Fluorocholine was administered by intravenous bolus injection.

### 2.3. PET/CT Imaging and Evaluation

Our institution’s dedicated PET/CT scanner (Discovery 710; GE Healthcare, Milwaukee, WI, USA) was used to perform the studies, with an extended field-of-view full-ring high-resolution Lutetium yttrium oxyorthosilicate (LYSO) PET component with a 128-slice spiral CT.

Image acquisition occurred for all patients 60 min after intravenous (i.v.) injection of the radiotracer. In patients with biochemical recurrence, an early dynamic [^18^F]Fluorocholine PET imaging (1–8 min post-injection) was also performed in one bed-position over the prostate bed in order to assess the radiotracer’s early perfusion pattern and override any urinary radioactivity in the bladder. In cases of indeterminate findings in standard whole-body PET/CT, a delayed acquisition (90–120 min post-injection) was obtained from that region. As a routine protocol in our department, all patients received a standard volume (250 mL) of sodium chloride i.v. infusion and 250–500 mL of oral fluid. Standard whole-body acquisitions were obtained from the base of the skull to the proximal thigh with an acquisition time per bed position of 2.5 min using time of flight mode. Images were reconstructed identically using the ordered-subsets expectation maximization algorithm (four iterations, 18 subsets), then subsequently using a post-reconstruction smoothing gaussian filter (4.0 mm full-width at half-maximum). A contrast-enhanced-CT (CE-CT) scan with a high beam tube-current modulation (120–330 mA, 0.6 s per rotation, 5.0 mm reconstructed section thickness, 0.5 mm overlap, 512 × 512 matrix, pitch index 1.5) was completed in all patients, either in [^18^F]Fluorocholine or in [^68^Ga]Ga-PSMA PET/CT. Low beam current modulation (80–120 mA) was used for the CT portion of the second PET/CT. Reformatted transverse, coronal, and sagittal views were analyzed for interpretation.

Two independent board-certified nuclear medicine physicians at our institution interpreted the imaging. They noted observed lesions and calculated the number of suspected lesions; any discrepancies were resolved by discussion between the physicians until a consensus was reached. The nuclear medicine physician reporting one type of radiotracer scan for a given patient was unaware of the results from the other type of radiotracer scan for the same patient. Advanced PET/CT software (AW-4.6; GE Medical Systems, Milwaukee, WI, USA) that allows PET, CT, and fusion PET/CT data to be viewed simultaneously was used for reading.

Suspicious lesions were evaluated both visually and semi-quantitatively. In cases of disseminated metastases, only the five most prominent lesions in different anatomical structures (i.e., prostate and surrounding region, skeletal, regional and distant lymph nodes, and organ metastases) were included. Any area of increased radiotracer uptake greater than background uptake and not attributable to physiologic areas was considered suspicious for malignancy. Being aware of the non-specific uptake of both tracers in non-malignant and inflammatory diseases, most of the abnormal lesions were correlated with histopathology or other imaging findings. The number, location and intensity of lesions quantified by maximum standard uptake values (SUV max), were recorded. Patients’ demographics, Gleason score, serum PSA level, tumor, nodes and metastases (TNM) stage, number of lesions in each category, lesion size and SUV max were recorded in a prepared Excel form. The charts were also reviewed to determine whether PET/CT imaging affected clinical management. Changes in treatment planning due to detected lesions on PET/CT imaging were noted.

### 2.4. Statistics

Descriptive statistics were performed using Excel 2010 (Microsoft Corporation, Redmond, WA, USA). Quantitative variables were described using mean ± standard deviation (SD) and qualitative variables were described as percentages (%). Paired sample *t*-tests were used to compare size and SUV max in concordant lesions detected by both modalities after confirming normal distribution of the variables. SPSS software version 21 (SPSS Inc., Chicago, IL, USA) was used for this analysis. A *p*-value less than 0.05 was considered significant for all tests.

## 3. Results

### 3.1. Study Cohort

The current study included 29 patients who met the inclusion criteria outlined above, and received both [^18^F]Fluorocholine and [^68^Ga]Ga-PSMA PET/CT imaging within a maximum of 30 days of each other without any specific changes in concomitant prostate cancer treatment in this interval. Of these patients, 11 (38%) were referred for staging and 18 (62%) were referred for restaging of biochemical recurrence. The mean patient age was 68.8 ± 7.4 years (range: 56–83 years). The mean time between scans was 7.45 ± 9.1 days, with the shortest time interval between [^18^F]Fluorocholine and [^68^ Ga]Ga-PSMA PET/CT scanning being one day and the maximum being 30 days. With respect to order of scanning, 19 patients received [^68^Ga]Ga-PSMA PET/CT scanning first, and 10 received [^18^F]Fluorocholine PET/CT as the primary modality.

Patients included had a median Gleason score of 8. The minimum Gleason score of an included patient was 7 and the maximum was 9. In the staging group, PSA values ranged from 5 to 81 ng/mL, with a mean of 32.2 ± 23.8 ng/mL. In the restaging group, PSA values ranged from 0.07 to 3133 ng/mL.

### 3.2. Lesion Based Findings

Overall, 148 lesions were detected, of which 105 (70.9%) lesions were congruent in both modalities. [^68^Ga]Ga-PSMA PET/CT exclusively detected 30 (20.3%) lesions while 13 (8.8%) lesions were solely detected by [^18^F]Fluorocholine PET/CT (Table 1). A similar pattern was observed specifically for lymph node lesions. For the skeletal lesions, approximately the same percent were detected exclusively by each modality: 9 (15%) with [^68^Ga]Ga-PSMA PET/CT versus 8 (13%) by [^18^F]Fluorocholine PET/CT and 43 (72%). For the prostate, prostate bed and seminal vesicles region, only one additional lesion was detected by [^68^Ga]Ga-PSMA. In contrast, all three hepatic lesions were only detected by [^68^Ga]Ga-PSMA (Table 1). [^68^Ga]Ga-PSMA imaging was overall superior in the number and SUV max of detected lesions.

### 3.3. Prostate, Prostate Bed, and Seminal Vesicles

Seven prostate lesions, six prostate bed lesions, and two seminal vesicle lesions were detected by both modalities. In one patient in the staging group, one prostate lesion was exclusively detected by [^68^Ga]Ga-PSMA PET/CT, with a size of 4.8 mm and an SUV max of 10.2. The scan interval was two days in this patient; however, as the seminal vesicles were also involved in this patient and detected by both modalities, the TNM staging was not influenced.

**Figure 1:**
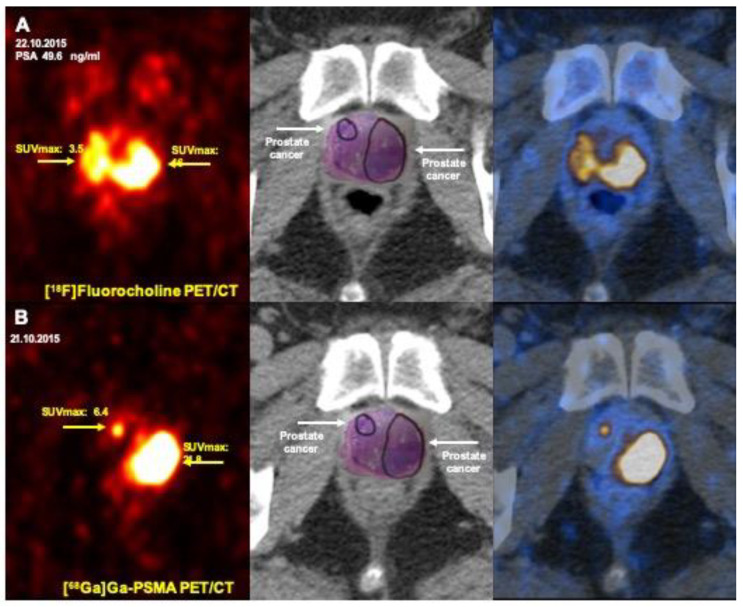
Prostate cancer staging: superior impact of [^68^Ga]Ga-PSMA PET/CT comparing to [^18^F]Fluorocholine PET/CT in the localization of primary tumor in the prostate (Gleason score 8, PSA: 49.6 ng/mL). (**A**) [^18^F]Fluorocholine PET/CT (transaxial PET (left), CT (middle), and fusion PET/CT (right) images) shows diffuse tracer uptake on both sides of prostate gland (arrows). A differentiation between inflammatory and malignant changes is not possible. (**B**) [^68^Ga]Ga-PSMA PET/CT (transaxial PET (left), CT (middle) and fusion PET/CT (right) images) shows an excellent correlation of [^68^Ga]Ga-PSMA and malignant changes in the prostate (arrows). PSA: Prostate Specific Antigen; SUV max: Maximum Standardized Uptake Value; PSMA: Prostate-Specific Membrane Antigen, PET/CT: Positron Emission Tomography/Computed Tomography.

In spite of concordance in overall number of lesions detected by both modalities, mean SUV max was significantly higher in lesions detected by [^68^Ga]Ga-PSMA compared to [^18^F]Fluorocholine PET/CT (14.6 ± 8.44 vs. 6.9 ± 3.4, *p*-value = 0.001; Table 2). In contrast to [^18^F]Fluorocholine PET, there was better correlation between [^68^Ga]Ga-PSMA PET and histopathological findings (Figure 1).

For [^68^Ga]Ga-PSMA PET/CT scanning, SUV max ranged from 4.9 to 28.5, with a mean SUV max of 14.6 ± 8.44. For [^18^F]Fluorocholine PET/CT scanning, SUV max of [^18^F]Fluorocholine lesions ranged from 2.8 to 16, with a mean SUV max of 6.9 ± 3.4.

### 3.4. Regional and Distant Lymph nodes

**Figure 2:**
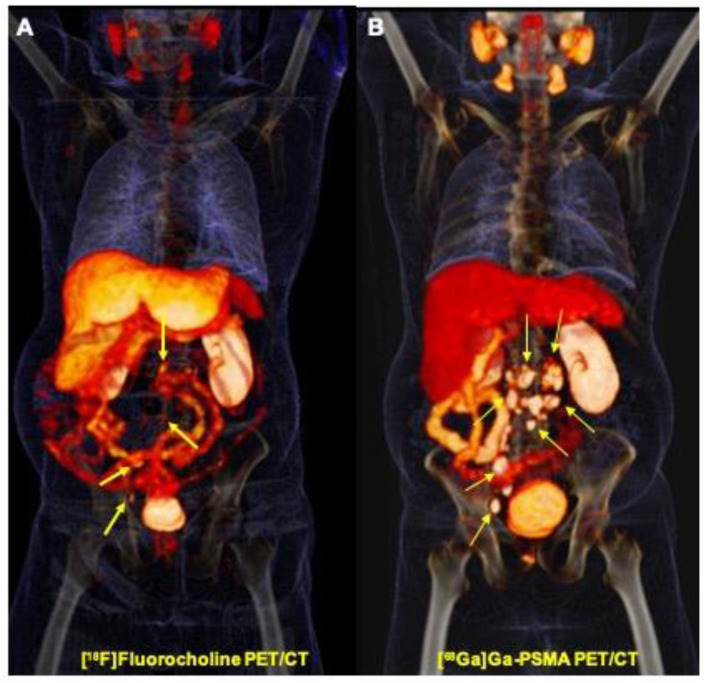
Prostate cancer recurrence: superior value of [^68^Ga]Ga-PSMA PET/CT comparing to [^18^F]Fluorocholine PET/CT in the detection of lymph node metastases (Gleason score 8, PSA: 25 ng/mL). (**A**) [^18^F]Fluorocholine PET/CT (volume-rendered maximum intensity projection (MIP)) shows multiple small faint [^18^F]Fluorocholine uptakes in the metastatic abdominal lymph nodes (arrows). (**B**) [^68^Ga]Ga-PSMA PET/CT (volume-rendered MIP) shows multiple intensive [^68^Ga]Ga-PSMA positive abdominal lymph nodes, clearly superior to [^18^F]Fluorocholine PET/CT (arrows).

Overall, 69 regional and distant lymph nodes were detected. Though the majority of lesions (47/69, 68.1%) were detected by both modalities, [^68^Ga]Ga-PSMA PET/CT detected significantly more lesions compared to [^18^F]Fluorocholine PET/CT (17 (24.6%) versus five (7.3%); Figure 2). SUV max was about three-fold higher in [^68^Ga]Ga-PSMA comparing to [^18^F]Fluorocholine PET/CT (20.3 ± 13.2 vs. 7.8 ± 4.1, *p*-value < 0.001; Table 2).

[^18^F]Fluorocholine PET/CT exclusively detected a right para-rectal lymph node, impacting the patient’s TNM score (Table 3). [^18^F]Fluorocholine PET/CT could detect some large additional lymph nodes in a castration resistant patient as well which will be further described in detail (Table 4).

[^68^Ga]Ga-PSMA PET/CT exclusively detected solitary lymphatic involvement in restaging of a patient with a PSA level lower than 0.5 ng/mL, leading to altered staging and management. Furthermore, [^68^Ga]Ga-PSMA PET/CT was significantly superior in detecting metastatic lymph nodes in two patients with PSA levels lower than 0.5 ng/mL and on hormone therapy.

Of the concordant lymph nodes, anatomic size ranged from 4.2 to 59 mm with a mean value of 16.2 ± 9.9 mm. However, [^68^Ga]Ga-PSMA PET uptake values by SUV max were significantly higher compared to [^18^F]Fluorocholine PET in concordant regional and distant lymph node metastases ([^68^Ga]Ga-PSMA PET mean SUV max: 20.3 ± 13.2, range: 3.2–62.9 vs. [^18^F]Fluorocholine PET/CT mean SUV max: 7.8 ± 4, range: 1.9–19.3; *p*-value= 0.000; Figure 2).

### 3.5. Skeletal Lesions

**Figure 3:**
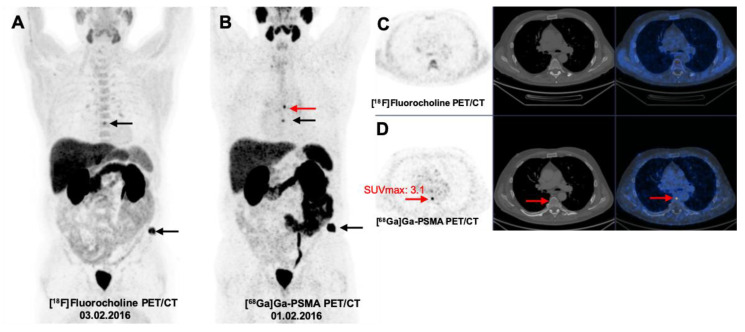
Prostate cancer recurrence: small skeletal metastases detected by [^68^Ga]Ga-PSMA PET/CT (Gleason score 8, PSA: 0.7 ng/mL). (**A**) [^18^F]Fluorocholine PET: maximum intensity projection (MIP) revealed multiple bone metastases (arrows) in the left ilium and T10 vertebra corresponding with the sclerotic changes on CT. (**B**) [^68^Ga]Ga-PSMA PET/CT: MIP detected additional small bone metastasis on T9 vertebra (red arrows) without relevant morphological changes on CT. (**C**) transaxial views: PET (left), CT (middle), and fusion PET/CT (right) images. (**D**) transaxial views: PET (left), CT (middle) and fusion PET/CT (right) images.

There were 60 bone lesions detected. The majority of the bone lesions (43/60, 71.6%) were detected by both modalities. Concordant lesions had a mean anatomic size of 17.3 ± 13.9 mm with a mean Hounsfield Unit (HU) of 594.3 ± 289.6. Although mean size of the lesions was not significantly different (19.8 ± 12.9 vs. 20.7 ± 13.4, *p*-value = 0.80), mean SUV max in [^68^Ga]Ga-PSMA PET/CT images were about two-fold higher than [^18^F]Fluorocholine PET/CT (23.4 ± 16.9 vs. 10.8±3.4, *p*-value < 0.001; Table 2, Figure 3).

[^18^F]Fluorocholine PET/CT was superior in exclusively eight bony lesions (8/60, 13%) in four patients (three restaging, one staging; Table 5).

**Figure 4:**
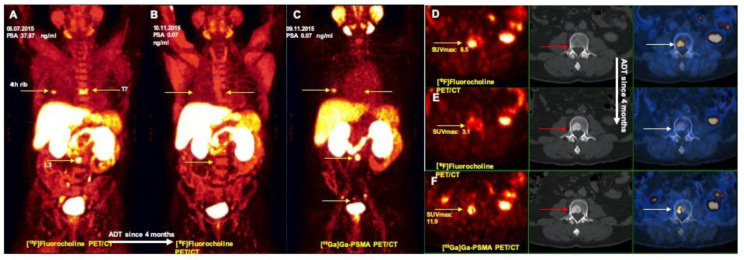
Prostate cancer treatment monitoring: more accurate evaluation of the treatment by [^68^Ga]Ga-PSMA PET/CT. (**A**) [^18^F]Fluorocholine PET: MIP revealed multiple bone metastases (arrows) on 4th right rib, T7 and L3 vertebra corresponding with morphological changes on CT. (**B**) Treatment monitoring by [^18^F]Fluorocholine PET: MIP showed no abnormal tracer uptake in the known metastases (arrows), suggestive complete response well correlating with PSA value of 0.07 ng/mL. (**C**) Treatment monitoring by [^68^Ga]Ga-PSMA PET MIP revealed still metabolic activity on the 4th right rib, and L3 vertebra (arrows) showing clear superiority to [^18^F]Fluorocholine PET and clinical findings. (**D**) transaxial views: PET (left), CT (middle) and fusion PET/CT (right) images. (**E**) transaxial views; PET (left), CT (middle) and fusion PET/CT (right) images. (**F**) transaxial views: PET (left), CT (middle) and fusion PET/CT (right) images.

**Figure 5:**
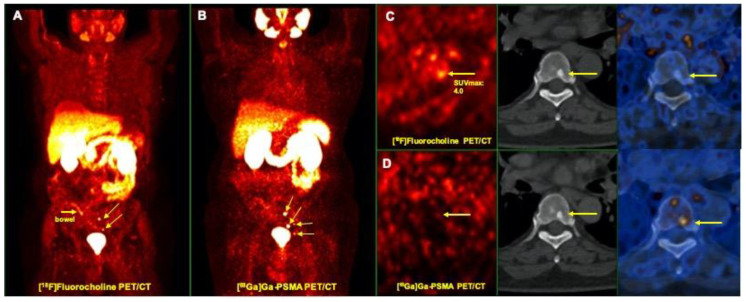
Prostate cancer recurrence: small skeletal metastases exclusively detected by [^18^F]Fluorocholine PET/CT. (**A**) [^18^F]Fluorocholine PET: MIP shows two small lymph nodes on the left pelvis suggestive of lymph node metastases (arrows). (**B**) [^68^Ga]Ga-PSMA PET/CT: MIP shows multiple intensive [^68^Ga]Ga-PSMA positive pelvic lymph nodes, clearly superior to [^18^F]Fluorocholine PET/CT (arrows). (**C**) [^18^F]Fluorocholine PET/CT (transaxial PET (left), CT (middle) and fusion PET/CT (right) images) exclusively detected a single bone metastasis on T6 vertebra well correlating with suspicious sclerosis on CT (arrows). (**D**) [^68^Ga]Ga-PSMA PET/CT (transaxial PET (left), CT (middle) and fusion PET/CT (right) images) shows limited value for detecting bone metastasis on T6 vertebra in spite of its superiority for detecting lymph node metastases.

Among the [^68^Ga]Ga-PSMA PET/CT superiority group, two (2/60, 3%) small sub-centimetric lesions were seen on the ilium with mild [^68^Ga]Ga-PSMA uptake. However, these lesions were confirmed as benign by magnetic resonance imaging. This pattern has already reported in previous studies and should be cautiously interpreted [23]. Seven lesions suspicious for metastasis (7/60, 11.6%) belonged to four patients; three patients (2/3 under hormone therapy) had multiple metastatic lesions, most of which were detected by both modalities and thus the discrepant lesions did not affect staging or management. On the other hand, one of the patients in this group with a low PSA level of 0.07 was considered to be in complete remission for bone lesions under hormone therapy according to [^18^F]Fluorocholine PET/CT, while three small bone lesions were detected exclusively by [^68^Ga]Ga-PSMA PET/CT (Figure 4). Interestingly, a small bone lesion was only detected by [^18^F]Fluorocholine PET/CT in a patient with additional sub-centimetric pelvic nodes on [^68^Ga]Ga-PSMA PET/CT (Figure 5).

### 3.6. Organ Metastases

Three hepatic metastases were detected only by [^68^Ga]Ga-PSMA PET/CT in a patient with a PSA level of 753 ng/mL and on hormone therapy.

### 3.7. Patient Based Findings

#### 3.7.1. TNM Staging

TNM staging and subsequently patient management was only affected by scanning in four of 29 patients (14%). Of these four patients in whom TNM staging was affected, one was referred for staging and three were referred for restaging (Table 3).

#### 3.7.2. Discordant Lesions

Discordant lesions were detected by both modalities, but more frequently by [^68^Ga]Ga-PSMA PET/CT, particularly in association with lower PSA values and hepatic metastases. [^18^F]Fluorocholine PET/CT exclusively detected 13.3% of skeletal and 7.3% of distant nodal involvements. The skeletal lesions found only on [^18^F]Fluorocholine PET/CT were not associated with lesion morphological characteristics on CT, including lytic/sclerotic nature and Hounsfield units. We were not able to contribute the discordant [^18^F]Fluorocholine PET/CT avid lesions with patients’ PSA values. Also, we found no correlation between Gleason Scores or ongoing Androgen-deprivation therapy (ADT) with morphological findings and Hounsfield units on CT. This lack of a relationship between discrepant findings and morphological patterns, as well as clinical data, has already been reported in the literature [24]. In spite of the limited number of patients, some patterns could be delineated from the discrepant [^18^F]Fluorocholine PET/CT-only positive lesions (Table 4 and Table 5).

#### 3.7.3. Correlation of PET Positivity and PSA Value

Overall lesion ratios detected by each modality were 91% for [^68^Ga]Ga-PSMA PET/CT and 78% for [^18^F]Fluorocholine PET/CT. In patients with PSA levels below 1.0 ng/mL, [^18^F]Fluorocholine PET/CT was able to detect only 57% of the lesions diagnosed by [^68^Ga]Ga-PSMA PET/CT (8/14 lesions). In PSA values < 2.0 ng/mL, the number of lesions detected by [^18^F]Fluorocholine PET/CT was half (55%) the number of lesions detected by [^68^Ga]Ga-PSMA PET/CT (11/20).

Moreover, in patients with low PSA values (under 0.5 ng/mL), [^68^Ga]Ga-PSMA PET/CT demonstrated not only remarkable superiority in lesion detection compared to [^18^F]Fluorocholine PET/CT, but also detected additional metastatic lesions that led to changes in treatment decisions (Figure 3 and Figure 4).

## 4. Discussion

The high associated mortality and recurrence rate of PC emphasizes the need for improved management strategies, thereby necessitating selection of the most accurate imaging techniques. In this study, we endeavored to compare the performance of PET/CT modality using the more novel radiotracer [^68^Ga]Ga-PSMA with [^18^F]Fluorocholine for the assessment of prostate cancer patients as both modalities are performed routinely in different centers. Reviewing the literature, few studies exist investigating the size and radioactivity values of both tracers in prostate cancer and directly comparing the two modalities in clinical staging and patient management [24,25,26].

In accordance with the previous studies [24,26], this article finds the performance of [^68^Ga]Ga-PSMA PET/CT to be overall superior to [^18^F]Fluorocholine PET/CT, particularly in patients with low PSA levels, small pelvic lymph nodes, small bone lesions, and hepatic metastases. In our study, although the majority of regional and distant lymph nodes were detected by both modalities, [^68^Ga]Ga-PSMA PET/CT detected significantly more lesions compared to [^18^F]Fluorocholine PET/CT (24.6% versus 7.3%, respectively). This was likely due to higher SUV max (approximately three times more) in [^68^Ga]Ga-PSMA compared to [^18^F]Fluorocholine PET/CT. Higher detection rates of [^68^Ga]Ga-PSMA PET/CT due to better lesion contrast compared with [^18^F]Fluorocholine PET/CT, especially in the low PSA level range, has also been reported by other authors [25,27]. This delineates [^68^Ga]Ga-PSMA PET/CT scanning as a more sensitive and accurate tool, particularly in early detection of local recurrences in patients with PSA levels <2.0 ng/mL.

[^68^Ga]Ga-PSMA PET/CT emerged as superior to [^18^F]Fluorocholine PET/CT in overall number of lesions detected, which is in line with other studies [25,27,28]. However, strikingly, there were lesions exclusively noted on [^18^F]Fluorocholine PET/CT, 13.3% being skeletal and 7.3% being in lymph nodes. In a previous study, Schwenck et al. analyzed the characteristics of discrepant lesions between the two modalities [24]. They studied 123 patients of primary and recurrent prostate cancer who were imaged by both [^68^Ga]Ga-PSMA and ^11^C-Choline PET/CT and found exclusive ^11^C-Choline PET/CT positive lesions to be 6% nodal and 2% skeletal lesions [24]. The number of skeletal exclusive [^18^F]Fluorocholine PET/CT positive lesions were significantly more in our investigation. This may be due to different nature of the two radiotracers (^11^C-Choline versus [^18^F]Fluorocholine) and different acquisition timing in the two studies (5 min acquisition time with an additional 20 min view of the pelvis with ^11^C-Choline in Schwenck et al. study versus 60 min acquisition time with [^18^F]Fluorocholine in our study). It also may be partly related to the aggressive tumor characteristics of our cohort, demonstrated by higher average SUV max and PSA values.

Schwenck and his colleagues reported that the discordant bone lesions had no significant correlation with the PSA levels [24]. They also postulated that the discrepancy in skeletal lesions may be correlated with the lesion size and/or castration resistant nature of the disease. Similarly, investigating the clinical data of our patients, we could not correlate discrepant [^18^F]Fluorocholine PET/CT positive skeletal lesions with PSA values, nor could we correlate them with morphological patterns on CT, including lytic/sclerotic morphology or Hounsfield units.

Additionally, in two castration refractory patients in our study population, [^18^F]Fluorocholine PET/CT was able to detect either markedly higher numbers of bone metastases or large extra lymph node involvement compared to [^68^Ga]Ga-PSMA PET/CT. It was previously reported that Androgen-deprivation therapy (ADT) didn’t negatively affect the detection rate (DR) of [^18^F]Fluorocholine PET/CT in PC patients with biochemical relapse [14,29]. Although Androgen-deprivation therapy may increase the over-expression of PSMA in patients with PC, long-term ADT may reduce the visibility of lesions on [^68^Ga]Ga-PSMA PET/CT. This may be the reason why the DR of [^18^F]Fluorocholine PET/CT was more than [^68^Ga]Ga-PSMA PET/CT in these two patients. Other possible explanations for the higher detection rate of [^18^F]Fluorocholine PET/CT compared to [^68^Ga]Ga-PSMA PET/CT include the superiority of [^18^F]Fluorocholine PET/CT in the detection of bone metastases in poorly differentiated prostate cancers, and its ability to detect extra nodes that have begun the process of dedifferentiation [30,31,32]. However, a clear conclusion cannot be drawn due to the limited number of patients and lack of histopathological examination of the metastases. Regarding discordant lymph nodes, Schwenck et al. suggested that if a lymph node was exclusively detected by [^68^Ga]Ga-PSMA PET/CT, it could be due to better lesion contrast on this imaging modality; and when a lesion was only detected on [^18^F]Fluorocholine PET/CT, it could partly be explained by non-specific [^18^F]Fluorocholine uptake in inflammatory nodes or by the variable biological characteristics of prostate cancer with different transporters being expressed. We also assume that discordant lymph node findings may be either due to increased radiotracer specificity and higher SUV max values on [^68^Ga]Ga-PSMA PET/CT, due to dedifferentiation in castration resistance patient, or due to non-specific tracer uptake in inflammatory nodes on [^18^F]Fluorocholine PET/CT. Although, [^68^Ga]Ga-PSMA PET/CT showed superior diagnostic performance, in two patients with widespread skeletal metastases detected by both modalities, few sub-centimetric lesions were missed on [^68^Ga]Ga-PSMA PET/CT. This could possibly result from significant [^68^Ga]Ga-PSMA uptake by larger metastases. Further studies are needed to explain this pattern.

Similar to the other studies, we also found a superior DR for [^68^Ga]Ga-PSMA PET/CT in hepatic metastases [25,33,34]. This may be due to higher background hepatic uptake on [^18^F]Fluorocholine PET/CT compared with [^68^Ga]Ga-PSMA PET/CT, which may mask the hepatic lesions. [^18^F]Fluorocholine reveals poor metabolic stability in vivo by being oxidized to [^18^F]Flurobetaine, mainly in renal and hepatic tissues [35]. Undesirable hepatic signals with [^18^F]Flurobetaine can interfere with target tumoral radiotracer uptake in late PET/CT images. The other reason may be the higher SUV max of [^68^Ga]Ga-PSMA lesions compared with [^18^F]Fluorocholine lesions, which could overwhelm the background liver uptake. However, it should be emphasized that although the majority of liver metastases in patients with prostate cancer are PSMA positive, some may be PSMA negative and therefore can be missed on [^68^Ga]Ga-PSMA PET/CT [36].

Despite the differences in the findings of the two modalities, TNM staging and subsequent patient management was not affected in the majority of patients in the current study (86%; Table 3). This contrasts the findings observed by Schwenck et al. They reported a 50% change in patients management using [^68^Ga]Ga-PSMA as opposed to [^18^F]Fluorocholine for PET/CT in biochemical recurrence stage [37].

We acknowledge some limitations to the current study. The small number of patients and retrospective study design limits what causal conclusions may be drawn, since no randomization or time-controlled interventions were possible. Additionally, the lack of histopathological verification as the gold standard in all discordant findings, especially in the skeleton, limits the analysis of these findings. However, it was not ethical to obtain biopsy for all lesions, especially when it wouldn’t affect patient management. Further prospective comparative studies are recommended in order to accurately evaluate the performance of these two radiotracers in the context of various tumor characteristics.

## 5. Conclusions

This comparative study demonstrates an overall superior performance of [^68^Ga]Ga-PSMA compared to [^18^F]Fluorocholine for PET/CT imaging in prostate cancer patients referred for staging and restaging. Such superiority was mostly notable in patients with low PSA levels, small sub-centimetric bone lesions, small pelvic lymph nodes, and hepatic involvement. However, [^18^F]Fluorocholine PET/CT was likely more useful in particular clinical conditions. [^18^F]Fluorocholine PET/CT detected additional bony lesions not seen on [^68^Ga]Ga-PSMA PET/CT, particularly in high-risk hormone resistant PC patients, suggesting poorly differentiated metastases.

## Figures and Tables

**Table 1:** Number of lesions detected by both and exclusively by each modality according to anatomical regions.

Modality	Anatomic Category				Overall Lesion Number
	Prostate, Prostate Bed and Seminal Vesicles	Skeletal	Regional and Distant Lymph Nodes	Soft Tissue (Hepatic)	
[^68^Ga]Ga-PSMA PET/CT positive only	1 (6.3%)	9 (15.1%)	17 (24.6%)	3 (100%)	30 (20.3%)
[^18^F]Fluorocholine PET/CT positive only	0	8 (13.3%)	5 (7.3%)	0	13 (8.8%)
Positive on both modalities	15 (93.7%)	43 (71.6%)	47 (68.1%)	0	105 (70.9%)
Total	16	60	69	3	148

[^68^Ga]Ga-PSMA PET/CT: 68-Gallium labelled prostate-specific membrane antigen Positron Emission Tomography/Computed Tomography.

**Table 2:** Mean SUV max of concordant lesions in each anatomic category according to type of imaging.

Anatomic Category			
Modality	Prostate, Prostate Bed and Seminal Vesicles SUV Max (Mean ± SD)	Skeletal SUV Max (Mean ± SD)	Regional and Distant Lymph Nodes SUV Max (Mean ± SD)
[^68^Ga]Ga-PSMA PET/CT	14.6 ± 8.4	23.4 ± 16.9	20.3 ± 13.2
[^18^F]Fluorocholine PET/CT	6.9 ± 3.4	10.8 ± 3.4	7.8 ± 4.1
*p*-value	0.001	<0.001	<0.001

SUV: Standardized Uptake Value; SD: Standard deviation.

**Table 3:** Patients with different TNM staging by two imaging modalities.

Patient Number	Referring Cause	PSA Level	Stage by [^68^Ga]Ga-PSMA PET/CT	Stage by [^18^F]Fluorocholine PET/CT
20	restaging	0.07	N1M1b	N1 (false)
3	restaging	0.35	N1, stage IV	Complete remission (false)
6	staging	21.4	T2c, stage IIB	N1, stage IV (false inflammatory node)
7	restaging	2.51	N1 (false)	N1M1b

TNM: Tumor, Nodes and Metastases; PSA: Prostatic Specific Antigen.

**Table 4:** Discrepant nodal metastases exclusively detected on [^18^F]Fluorocholine PET/CT; 7.3% of all nodal involvements.

Patient Number	Clinical History	Imaging Findings	Note
13	Referred for Staging PSA = 61 Gleason score = 7 castration resistant (hormone therapy, chemotherapy, local radiation).	In Prostate bed and skeleton the [^68^Ga]Ga-PSMA PET/CT was the superior imaging modality. While variable pattern of imaging superiority was found in lymph nodes lesions, [^18^F]Fluorocholine PET/CT was prominent in some prominent nodes, even up to 60 mm in size.	Castration refractory prostate cancer and elevated PSA value not responding to conventional treatments; [^18^F]Fluorocholine PET/CT superiority may belong to dedifferentiating nodal metastases.
6	Referred for Staging PSA = 21.42 Gleason Score = 8 (TURP performed. After two months PSA reaches 0.17 in one year follow up).	In Prostate gland, [^68^Ga]Ga-PSMA PET/CT was the superior modality. No skeletal lesion detected. There is a solitary [^18^F]Fluorocholine positive node in right para-rectal space (measuring 5 mm, node SUV max 2.19).	PSA reached its nadir (0.17) after TURP and the node was assumed inflammatory pelvic node. By considering borderline radioactivity SUV max 2.19, we may avoid misinterpretation.

SUV: Standardized Uptake Value; PSA: Prostatic Specific Antigen; TURP: Transurethral Radical Prostatectomy.

**Table 5:** Discrepant skeletal lesions exclusively detected by [^18^F]Fluorocholine PET/CT; 13.3% of all skeletal involvements.

Patient Number	Clinical History	Imaging Findings	Note
17	Referred for Staging PSA = 81.17 Gleason Score = 7	In Prostate bed and lymph nodes [^68^Ga]Ga-PSMA PET/CT was the superior imaging modality; While variable pattern of imaging superiority was found in skeletal lesions, [^18^F]Fluorocholine PET/CT was prominent in some skeletal lesions including small and negligible lesions in L2 vertebra(measured 10 mm) and left 8th rib (measured 8 mm)	[^18^F]Fluorocholine PET/CT superiority was only in few, small and sub-centimetric lesions may be due to significant [^68^Ga]Ga-PSMA uptake by larger metastases.
29	Referred for RestagingPSA = 19.3 Gleason Score = 8 Progressive disease	While variable pattern of imaging superiority was found in skeletal lesions, [^18^F]Fluorocholine PET/CT was prominent in some skeletal lesions including small and negligible lesions measuring up to 17 mm.	[^18^F]Fluorocholine PET/CT superiority was only in few, small and sub-centimetric lesions may be due to significant [^68^Ga]Ga-PSMA uptake by larger metastases.
5	Referred for Restaging PSA = 2078 Gleason score = 9 Castration resistant progressive disease (Xtandi, Zytiga, chemotherapy with Zyklen and Taxotere)	No prostate lesion or lymphatic involvement detected in the patient. Multiple bone lesions were noticed; all prominent in number and SUV (twice) in [^18^F]Fluorocholine PET/CT.	Castration refractory prostate cancer and elevated PSA value (2078) not responding to conventional treatments; [^18^F]Fluorocholine PET/CT superiority may be due to dedifferentiating metastases.
7	Referred for Restaging PSA = 2.51ng/mL previous PSA one month before was 5ng/mL Gleason score = 9	No prostate bed or soft tissue involvement detected by the imaging modalities, The two lymph nodes detected were prominent in [^68^Ga]Ga-PSMA scan. Bone lesions (7 mm in T11 and 12 mm in T6 vertebra) were exclusively detected by [^18^F]Fluorocholine PET/CT and confirmed by MRI.	A pattern could not be supposed explaining small vertebral metastases exclusively found on [^18^F]Fluorocholine PET/CT (confirmed by MRI).

SUV: Standardized Uptake Value; PSA: Prostatic Specific Antigen; MRI: Magnetic Resonance Imaging.
